# Phenotypic, genotypic and antigenic characterization of emerging avian reoviruses isolated from clinical cases of arthritis in broilers in Saskatchewan, Canada

**DOI:** 10.1038/s41598-017-02743-8

**Published:** 2017-06-15

**Authors:** Lisanework E. Ayalew, Ashish Gupta, Jenny Fricke, Khawaja Ashfaque Ahmed, Shelly Popowich, Betty Lockerbie, Suresh K. Tikoo, Davor Ojkic, Susantha Gomis

**Affiliations:** 1 Veterinary Pathology, Western College of Veterinary Medicine, University of Saskatchewan, Saskatoon, Saskatchewan Canada; 2 Vaccinology & Immunotherapeutic Program, School of Public Health, University of Saskatchewan, Saskatoon, Saskatchewan Canada; 3 Animal Health Laboratory, Laboratory Services Division, University of Guelph, Guelph, Ontario Canada

## Abstract

In recent years, emerging strains of pathogenic arthrogenic avian reovirus (ARV) have become a challenge to the chicken industry across USA and Canada causing significant economic impact. In this study, we characterized emerging variant ARV strains and examined their genetic and antigenic relationship with reference strains. We isolated 37 emerging variant ARV strains from tendons of broiler chickens with clinical cases of arthritis/tenosynovitis at commercial farms in Saskatchewan, Canada. Viral characterization using immunocytochemistry, gold-immunolabeling and electron microscopy revealed distinct features characteristic of ARV. Polymerase chain reaction-restriction fragment length polymorphism (PCR-RFLP) analyses of the viral Sigma C gene revealed genetic heterogeneity between the field isolates. On phylogenetic analyses, the Sigma C amino acid sequences of the isolates were clustered into four distinct genotypic groups. These ARV field strains were genetically diverse and quite distant from the vaccine and vaccine related field strains. Antibodies produced against a commercial Reo 2177 ^®^ vaccine did not neutralize these variants. Moreover, structure based analysis of the Sigma C protein revealed significant antigenic variability between the cluster groups and the vaccine strains. To the best of our knowledge, this is the first report on the genetic, phenotypic and antigenic characterization of emerging ARVs in Canada.

## Introduction

Avian reoviruses (ARVs) are divergent in their pathogenicity and can infect a variety of avian species. Chickens can be infected by several pathogenic avian reovirus (ARV) antigenic types, which are associated with various disease conditions including viral arthritis/tenosynovitis, stunting/malabsorption syndrome, pericarditis, myocarditis, hepatitis, respiratory and enteric diseases. The direct cause and effect association has not been conclusively determined except for viral arthritis/tenosynovitis syndrome^1^. ARV is the major cause of viral arthritis/tenosynovitis in young broiler chickens affecting weight bearing joints. The disease is characterized by swelling of the foot-pad and the hock joint, which leads to lameness. Depending on the degree of severity, the affected birds may be unable to walk resulting in poor growth, poor production and sometimes death^2, 3^. Emerging ARVs can cause up to 10% mortality and 20–40% morbidity in broiler chickens, which may result in significant economic losses^4^.

ARVs are RNA viruses with a non-enveloped icosahedral capsid with 10 double stranded genome segments^5^. ARV belongs to the *Orthoreovirus* genus in the family *Reoviridae* ^6, 7^. The genomic segments are divided into three size classes (i.e. Large [L], Medium [M] and Small [S]) based on their electrophoretic mobility on a polyacrylamide gel^8^. The ARV genome encodes four non-structural proteins (μNS, μNS, P10 and P17) and eight structural proteins (λA, λB, λC, μA, μBC, σA, σB and σC)^9^. The σ (Sigma) C protein is expressed by the third open reading frame of the S1 gene. The protein is 326 amino acids long and is the most variable protein in the reovirus genome^10^. The protein contains both type specific and broadly specific epitopes, and induces the production of neutralizing antibodies^11^. Hence, the Sigma C sequence is used as a genetic marker to characterize and classify reovirus isolates into different genotype/pathotype groups.

In recent years, the Saskatchewan broiler industry has seen an increased incidence of reovirus-associated arthritis/tenosynovitis despite vaccination programs against ARVs. This may indicate that vaccination programs are not properly adhered to or that field ARVs are not amenable to current commercial vaccines. Besides, there is a lack of data regarding the status of ARVs currently circulating in Canada. Therefore, the objective of this study was to isolate and characterize the emerging ARVs capable of causing viral arthritis/tenosynovitis in broiler chickens by escaping immunity induced by conventional commercial ARV vaccines in the Saskatchewan broiler industry.

## Results

### Sero-prevalence of ARV

**Figure 1:**
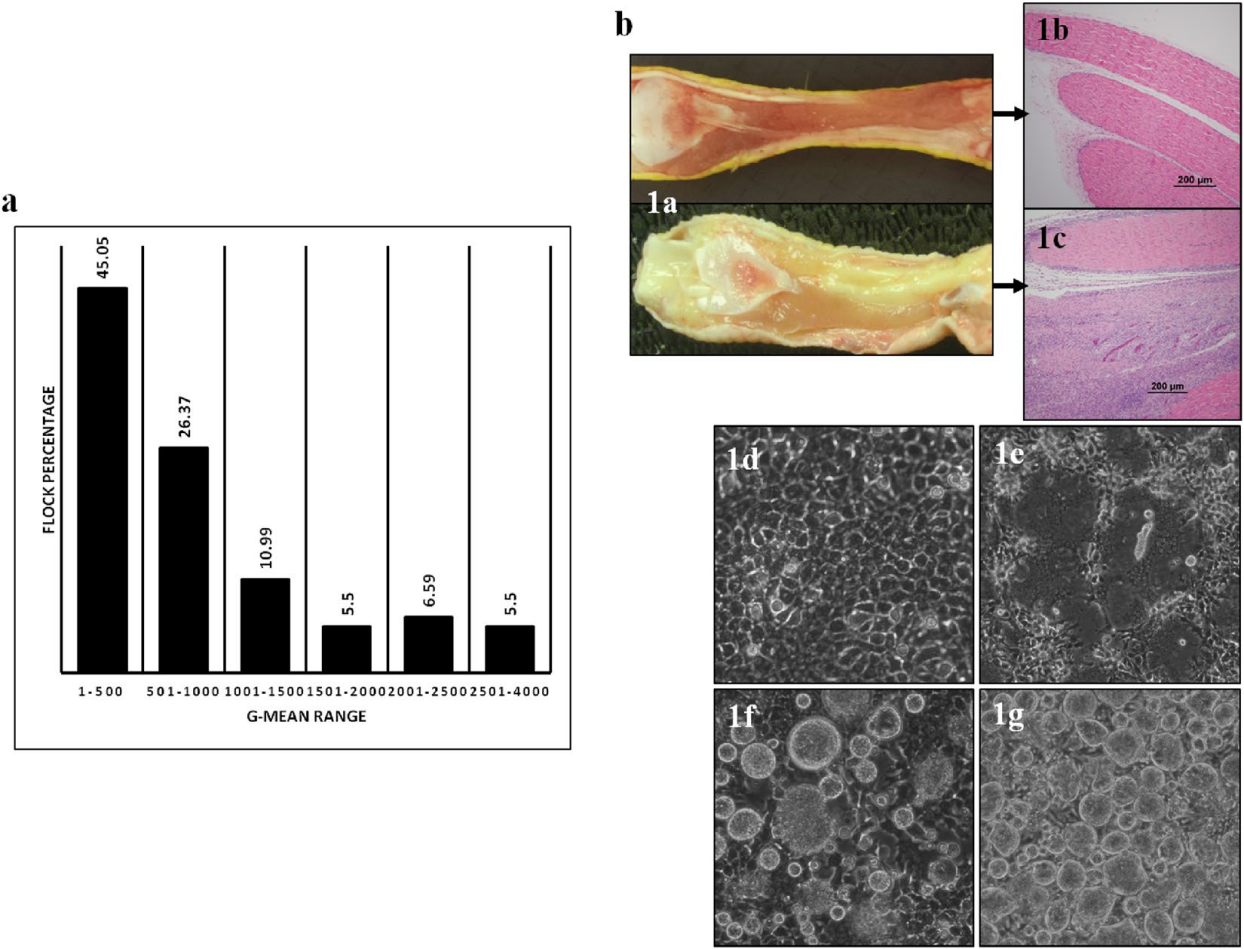
(**a**) The frequency distribution of ARV ELISA geometric means of 59 farms. (**b**) Gross and histopathological lesions in ARV infected tendon tissue, and ARV induced CPE in cell culture. (Panel a) ARV infected and non-infected tendon tissues from broiler chickens. (Panel b) Histology of normal tendon tissue. (Panel c) Lymphocytic plasmacytic infiltration in ARV infected tendon tissue with moderate number of hetrophils. (Panel d) Mock infected LMH cells. Panels (e) (f) and (g) 18, 24 and 36 hr post- infection of LMH cells with an ARV isolate, respectively.

A survey of the prevalence of ARV was conducted at 59 of 63 (94%) broiler chicken farms in Saskatchewan, Canada. Based on the ARV ELISA test, 98.3% of the farms were positive (mean ELISA cut-point 396, IDEXX reference guide) with the percentage of birds with arthritis ranging from 10 to 15% between flocks at the time of blood sample collection. Since The IDEXX ELISA kit plates are coated with a whole virus lysate, the kit is not strain specific and detects a wide range of pathogenic and non-pathogenic strains, therefore antibody level and disease are not directly associated. The ELISA geometric mean titers ranged between 149 and 3,759 with minimum and maximum individual titers of 0 and 35,840, respectively. The frequency distribution of G-mean ELISA titers of the flocks is shown in Fig. 1a.

### Clinical signs, necropsy and histopathologic lesions

ARV infected birds showed unilateral or bilateral inflammation of tendons (Fig. 1b, panel 1a). Ruffled feathers, lameness, splayed legs and reluctant to get up and walk were the main clinical signs observed. Histopathologic examination was performed on tendon tissues suspected of ARV infection to characterize the type of microscopic lesions. Unlike tendons from healthy birds (Fig. 1b, panel 1b), the tendon sheaths of infected birds were thickened with infiltration with mononuclear cells, chiefly lymphocytes, plasma cells and moderate number of heterophils at the site of active inflammation (Fig. 1b, panel 1c). Lymphocytes infiltrated into dense aggregates in multifocal areas or diffusely through the entire length of the tendon sheath. Increased fibroplasia and collagen deposition were also common observations.

### Virus isolation and characterization from tendons of chickens with arthritis/tenosynovitis

**Figure 2:**
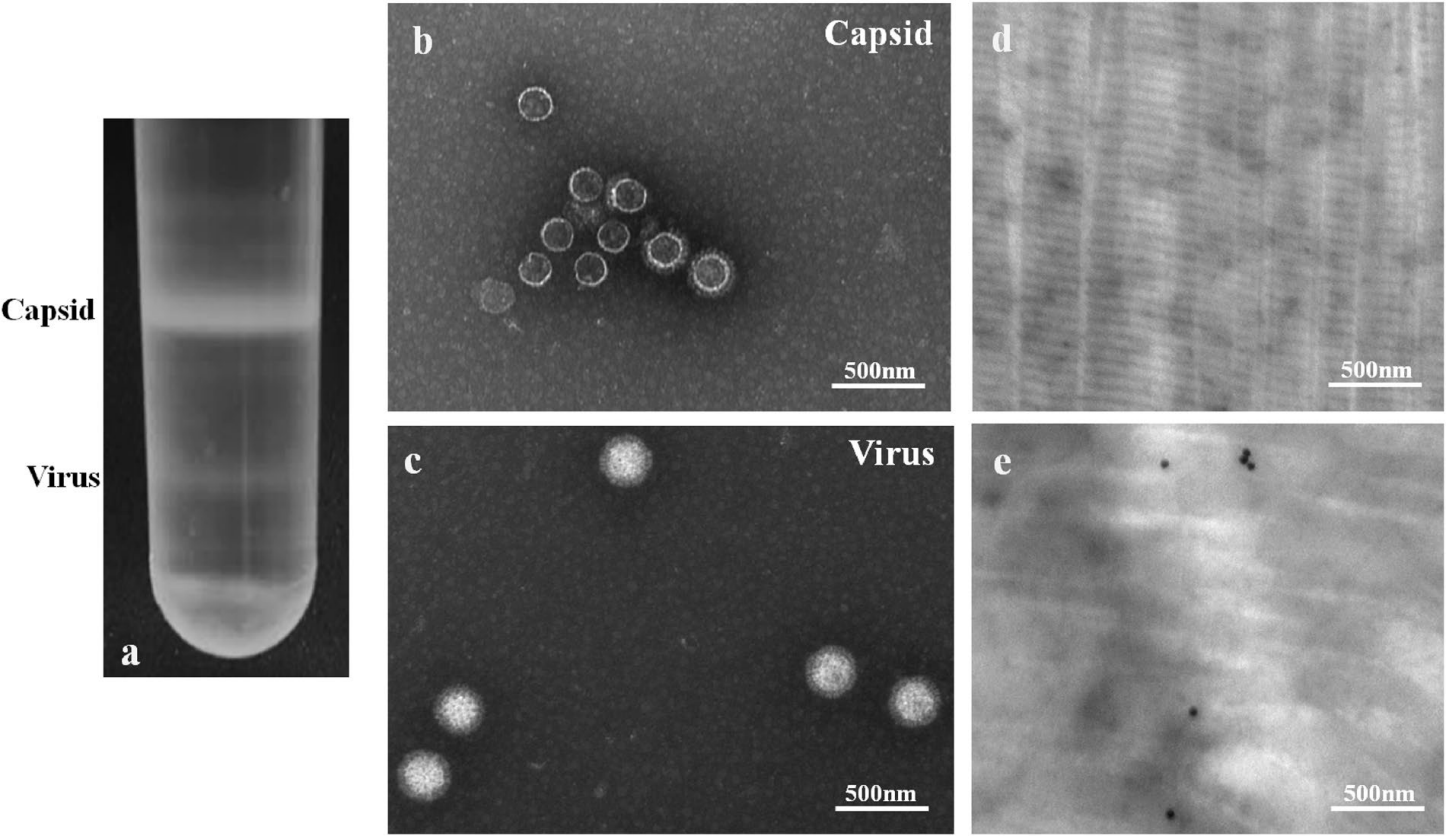
Virus purification and TEM, as well as ultrathin sectioning of tendon tissues and immunogold labelling TEM. (**a**) CsCl density gradient purification of ARV virus isolate showing bands of mature virions and empty capsids. (**b**) Negative staining TEM of purified empty capsids. (**c**) Negative staining TEM of mature ARV particles. (**d**) and (**e**) Ultrathin sectioning and immunoglod labeling using anti-ARV antibody of non-infected (d) and ARV infected (**e**) tendon tissues after negative staining TEM.

**Figure 3:**
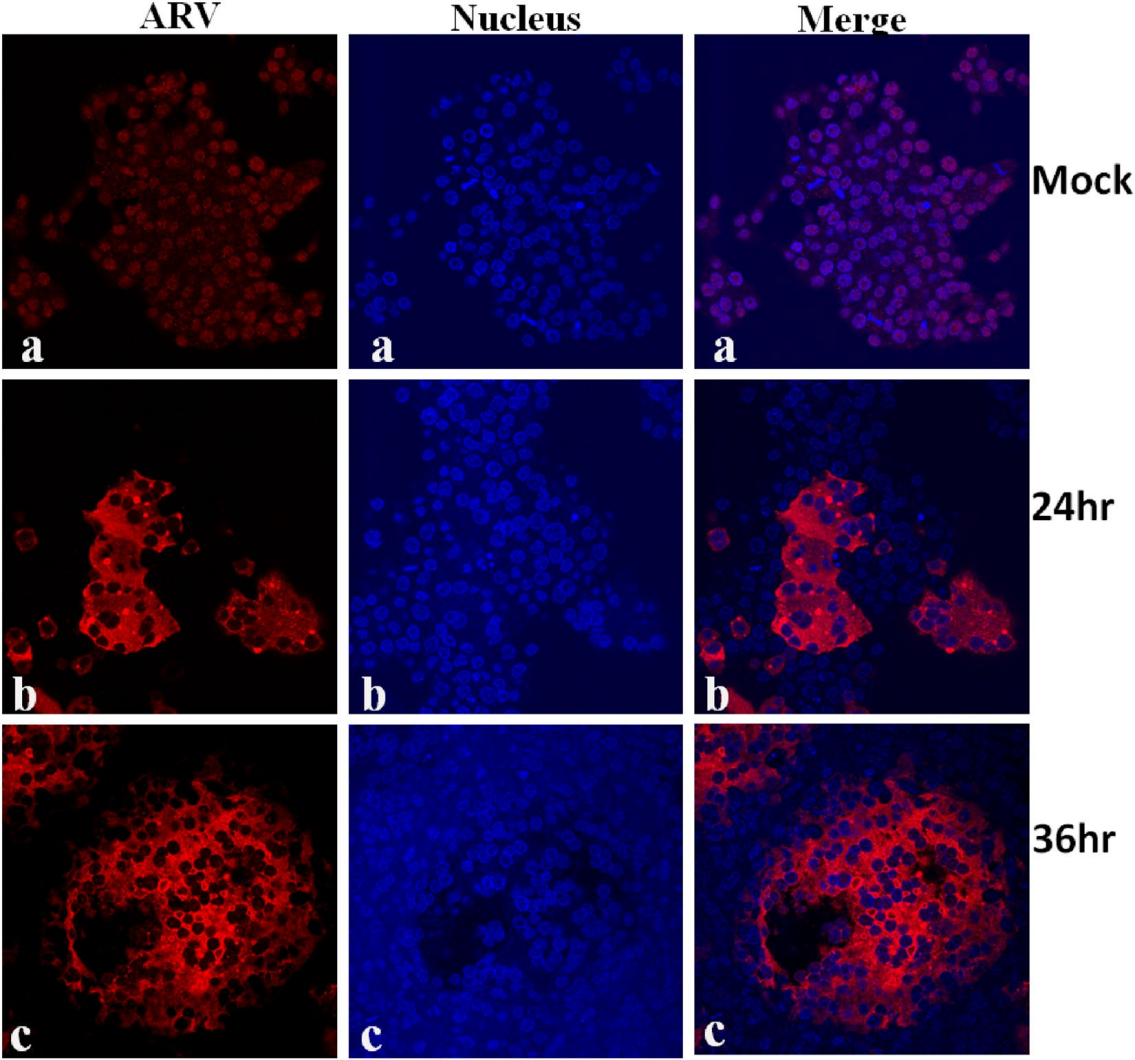
Indirect immunostaining of mock or virus infected cells using anti ARV primary antibody and AlexaFlour 647 conjugated secondary antibody. (Panel a) mock infected LMH cells. (Panels b and c) 24 hr and 36 hr post infection of LMH cells with virus isolate, respectively. DAPI was used as a nuclear counter stain.

To isolate virus, tendon samples of broiler chickens that demonstrated clinical signs of lameness/tenosynovitis were collected from broiler farms in Saskatchewan, Canada. The reovirus positive samples demonstrated a typical reovirus CPE with fusion of cells and formation of syncytia in cell culture (Fig. 1b, panel 1e). In all the positive cases, CPE was observed on the first passage. At later times post infection, the fused cells detached from the surface of the cell culture plate (Fig. 1b, panel 1f and g). No such change was observed in the cells inoculated with reovirus negative samples (Fig. 1, panel1d). Overall, a total of 37 ARVs were isolated (Supplement Table 1) and 82.05% of the samples were positive. To further characterize the isolates, virus purification was performed by CsCl density gradient centrifugation from infected cell lysates. As shown in Fig. 2a, clear bands of mature virions and empty capsid were effectively separated. Negative staining and TEM of CsCl purified empty capsids (Fig. 2b) or mature virions (Fig. 2c) demonstrated a structure with double capsid layers with large spikes/torrets projecting at the vertices of the core particle. Ultrathin sectioned, immuogold labelled tendon tissues from cases of arthritis/tenosynovitis using anti-ARV antibodies revealed high contrast dark spots on negative staining TEM (Fig. 2e). No such spots were observed on negative control samples (Fig. 2d). In addition, indirect immunofluorescence staining of the cells and confocal microscopy using anti-reovirus polyclonal antibody specifically detected reovirus proteins at 24 hr (Fig. 3, panel b) and 36 hr (Fig. 3, panel c) post-infection. No such protein was detected in mock infected negative controls (Fig. 3, panel a).

### Restriction fragment length polymorphism (RFLP) profiles of ARV isolates

**Figure 4:**
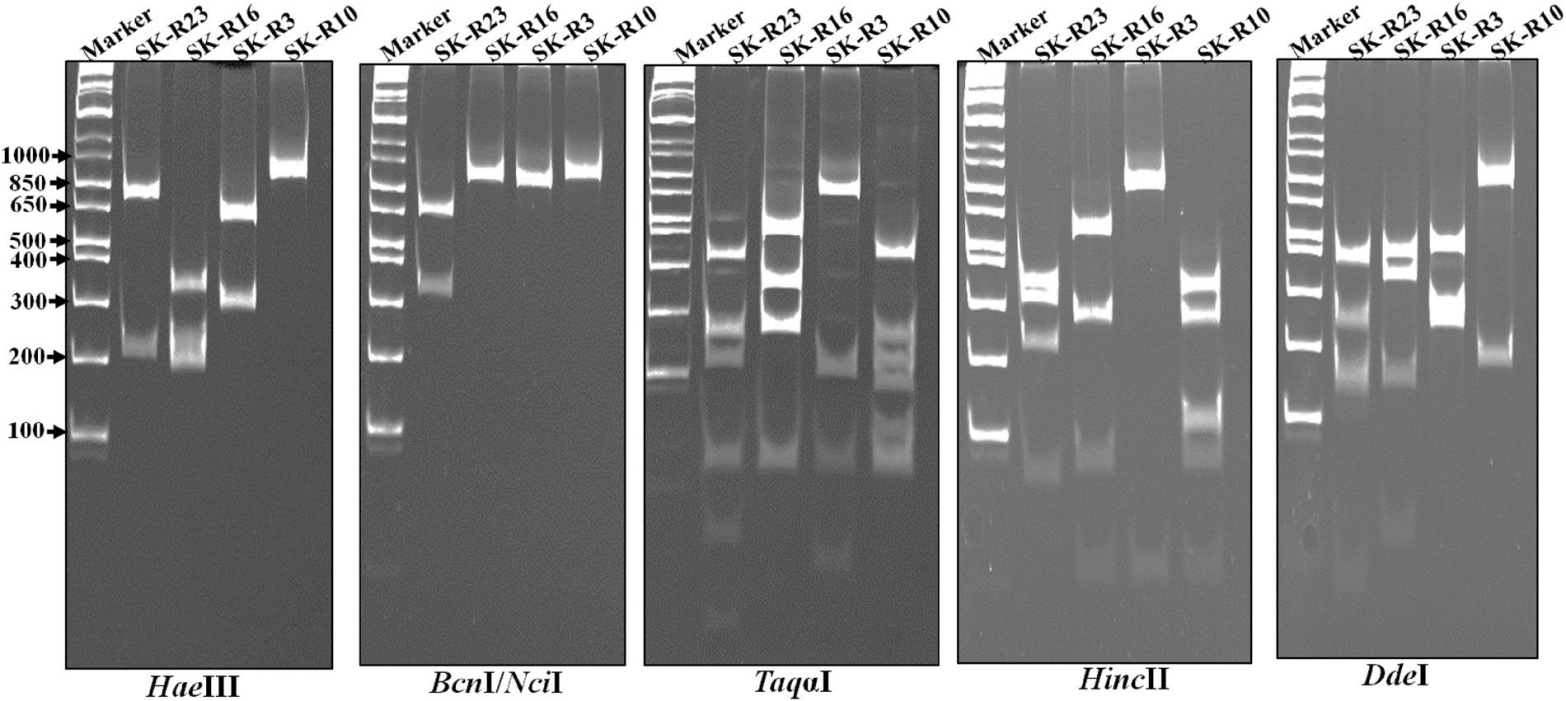
Polyacrylamide gel electrophoresis of the Sigma C RT-PCR products (~960 bp) following digestion with the indicated restriction enzymes. The DNA molecular weight markers are indicated on the left.

The RFLP profiles of our isolates were determined by digesting the RT-PCR amplified Sigma C gene fragments with various restriction enzymes^12^. Figure 4 shows the RFLP profiles of Sigma C gene fragments as obtained using *Hae*III, *Bcn*I, *Taq*Iα, *Hinc*II and *Dde*I restriction enzymes. As seen in (Fig. 4), the DNA polyacrylamide gel electrophoresis results revealed different cleavage patterns suggesting heterogeneity among the field ARVs.

### Sequence and phylogenetic tree analysis of the ARV isolates

**Figure 5:**
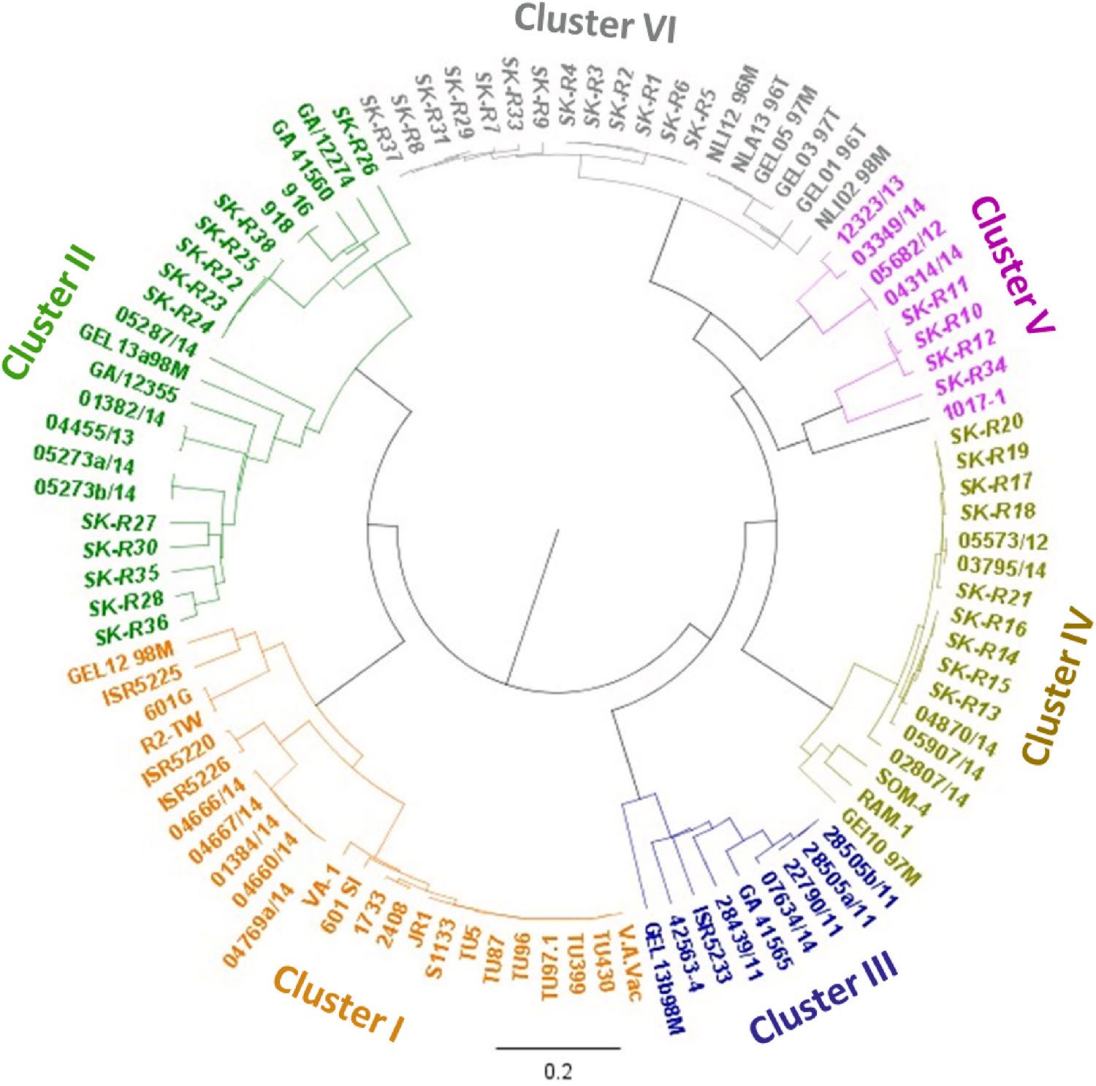
Phylogenetic tree of ARV strains based on the Sigma C sequence variability. The tree shows genetic relationship between the Sigma C protein sequence of 37 field isolates and 63 reference and variant strains isolated from around the world. The virus strains clustered into six genotyping groups (color coded). Our isolates are highlighted in bold. The tree was built by Neighbor joining method with Jukes-Cantor genetic distance model.

**Figure 6:**
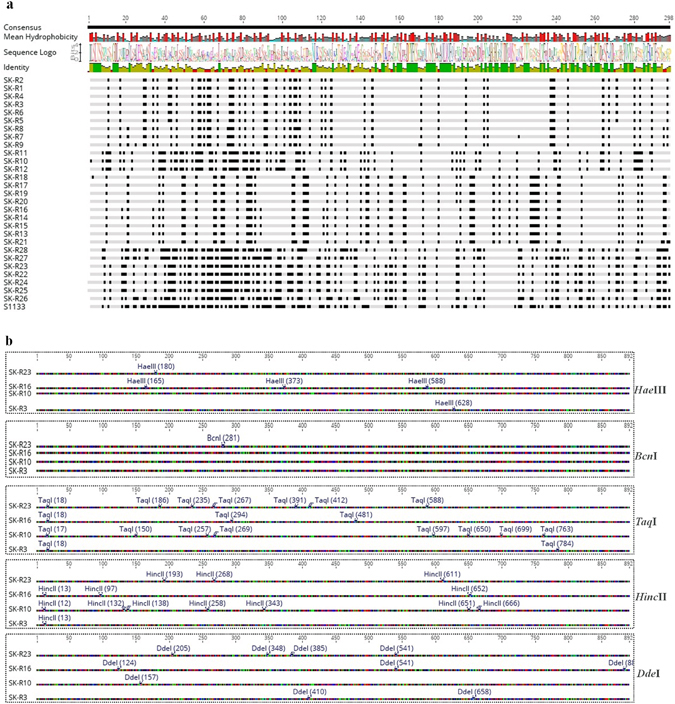
(**a**) A multiple amino acid sequence alignment of the common American vaccine strain S1133 and the 28 ARV field isolates. The alignment was made by a ClustalW alignment with BLOSUM cost matrix technique. Disagreements to the consensus sequence are highlighted. (**b**) Mapping of *Dde*I, *Hinc*II, *Taq*aI, *Bcn*I and *Hae*III restriction sites on Sigma C sequences of the ARV isolates (i.e. one isolate from each cluster group SK-R23 [Cluster II], SK-R16 [Cluster IV], SK-R10 [Cluster V], SK-R3 [Cluster VI]).

Since Sigma C gene of ARV is the most variable region in the viral genome, it is used to classify reovirus isolates into different genotyping groups^10^. To classify and study the genotypic properties of the ARV isolates, the Sigma C gene of each isolate was sequenced. The phylogenetic tree construction and analysis was performed based on the amino acid sequence of the Sigma C protein of our isolates (Supplement Table 1) alongside the Sigma C protein sequences of 63 reference strains and variants isolated from other parts of the world which were retrieved from GenBank (Supplement Table 2). In general, all of the viruses included in the study were grouped into six genotyping groups (Fig. 5). Our isolates were grouped into four distinct genotyping clusters (Clusters II, IV, V and VI). Surprisingly, none of the isolates were clustered together with the reference vaccine strains. Moreover, none of the isolates from the year 2013 were grouped under cluster IV. In addition, none of our isolates were clustered under cluster III. The amino acid sequence identity between S1133 (a commonly used vaccine strain) and our isolates was analyzed after multi-paired alignment of the sequences using Blosum ClustalW alignment program on Geneious 9.1.5 software. As shown in Fig. 6a, the amino acid sequence disagreements with the consensus sequence are highlighted. The sequence identity (i.e. percentage of amino acid residues which were identical) among our isolates was between 48.148 to 100%. The identity between the sequences clustered in groups II, IV, V and VI varied between 63.082 to 100%, 97.306 to 98.316%, 77.778 to 100% and 94.613 to 100%, respectively. The sequence identity between the S1133 vaccine strain and our isolates ranged between 46.465 to 54.209%. Eleven isolates grouped together with ARVs isolated from Taiwan (916 and 918 strains), USA (GA/12355 and GA/12274) and Germany (GEL 13a98M) in Cluster II showed a sequence identity ranging between 67.5 to 85.5%, 64 to 86% and 66.8 to 75%, respectively. In addition, the recent US Turkey reovirus isolates^4^ had a maximum sequence identity ranging from 65.614 to 83.509% with our isolates under Cluster-II (Supplement Figure 2). Nine isolates were sub-clustered with German (GEI10 97M)^13^ and Australia (SOM-4 and RAM-1) isolates^14^ under Cluster IV and shared 80.8 to 82% and 80 to 84% sequence identity, respectively. Four isolates grouped under Cluster V with a Taiwan (1017–1) isolate shared a sequence identity ranging from 68 to 69%. The rest of the isolates sub-clustered separately under Cluster VI with isolates from Germany (GEL01 96T, GEL03 97T, GEL05 97M) and the Netherlands (NLA13 96T, NLI12 96M, NLI02 98 M)^13^ sharing a sequence identity ranging from 87 to 90% and 88 to 90%, respectively. The Sigma C protein sequence of the most recently isolated ARV strains from the USA^4^ and our isolates had a 63.303 to 88.8%, 94.737 to 99.298% and 58.246 to 60% sequence identity with the isolates that grouped under Clusters II, IV and V, respectively. As shown in Fig. 6b, the *Bcn*I enzyme cutting site was only present in the sequence of the SK-R23 isolate from Cluster II (Fig. 5) creating two DNA bands after digestion of the Sigma C PCR product (Fig. 4). On the contrary, *Hae*III, *Hinc*II, *Dde*I and *Taq*Iα produced different cleavage patterns for the isolates from the different cluster groups. All Sigma C sequences from each genotyping group had similar restriction enzyme map based on the selected enzyme sites, but different between the cluster groups (Supplement Figure 1). In addition, a multiple alignment of the Sigma B protein sequences of our isolates and the Sigma B protein of the S1133 vaccine strain revealed a 92 to 100% sequence identity (Supplement Figure 3).

### ARV vaccine strain 2177 induced antibodies were unable to neutralize the newly isolated variant strains

**Figure 7:**
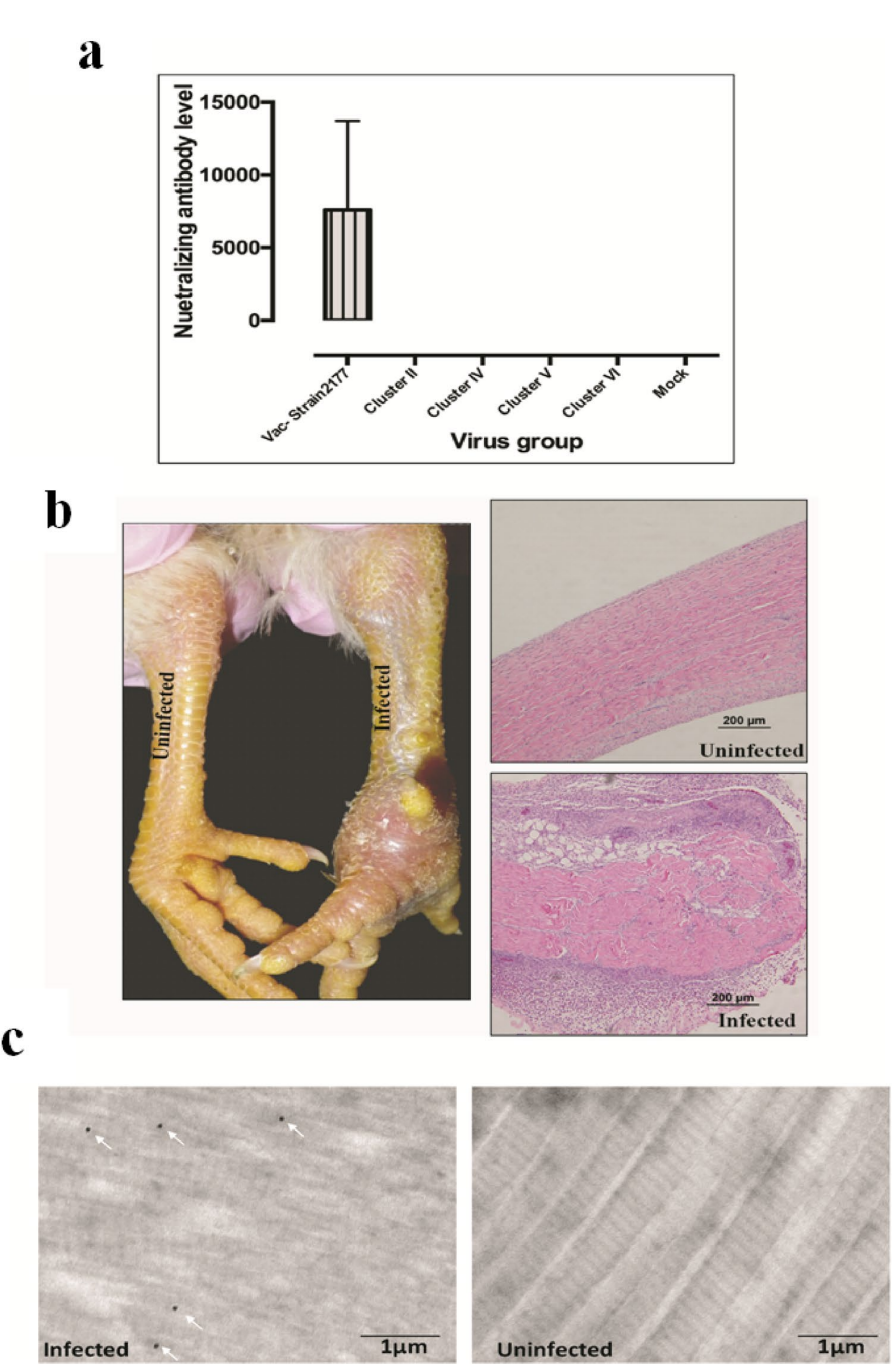
(**a**) Virus neutralization test. The neutralizing ability of antibodies produced against vaccine strain 2177 was tested against our field isolates from Cluster II, IV, V and VI. The vaccine virus strain 2177- antibody mixture and mock infected cells were used as positive and negative controls, respectively. The experiment was done in triplicates. (**b**) Gross and histopathological lesions of tendons from experimentally infected SPF birds. (**c**) Ultrathin sectioning and immunoglod labeling of tendon tissues using anti-ARV antibody from infected and non-infected SPF birds. The white arrows indicate gold labelled ARV particles.

Broiler breeder parent flocks in Saskatchewan (i.e. parent stocks of the broiler chickens where tendon samples were collected for ARV isolation) practiced ARV vaccination programs using commercially available vaccines (mostly Reo 2177 ^®^, Merck). However, in recent years disease symptoms suggestive of ARV infections have been increasing at several commercial broiler farms. To examine the virus neutralizing ability of the antibodies raised against the reovirus vaccine, homologous and heterologous VNT was performed in cell culture using sera from vaccinated birds having an average ELISA titer of 12,000. One reovirus isolate from each of the four cluster groups and the vaccine strain 2177 were tested. Except for the vaccine strain, none of our ARV isolates tested were neutralized (Fig. 7a).

### The ARV isolates from all the Cluster groups are virulent

All the ARV strains from each cluster group were capable of causing clinical disease. The disease, in all cases, was initially characterized by swelling of the foot pad which later extended to the tarsometatarsal joint. After 48 hrs post infection, all infected birds were inactive with ruffled feathers and were observed in a sitting position. When stimulated to react, they demonstrated lameness and some were reluctant to move. At necropsy, inflammation of the foot pad extending up to the hock joint was observed including the synovial membranes and the surrounding tissue. No significant lesions were observed in internal organs. Based on gross lesions, there was no significant difference in the severity of the disease induced by the different ARV strains. On histopathology, As compared to tendons from healthy birds, the tendon sheaths of infected birds were thickened with lymphocytic-plasmacytic infiltration (Fig. 7b). At later stages post infection, increased fibroplasia and collagen deposition were observed. In addition, virus was detected in the tendon tissues from all infected groups by immunogold-staining electron microscopy, but not from negative controls (Fig. 7c).

### Structure modelling and antigenicity prediction

**Figure 8:**
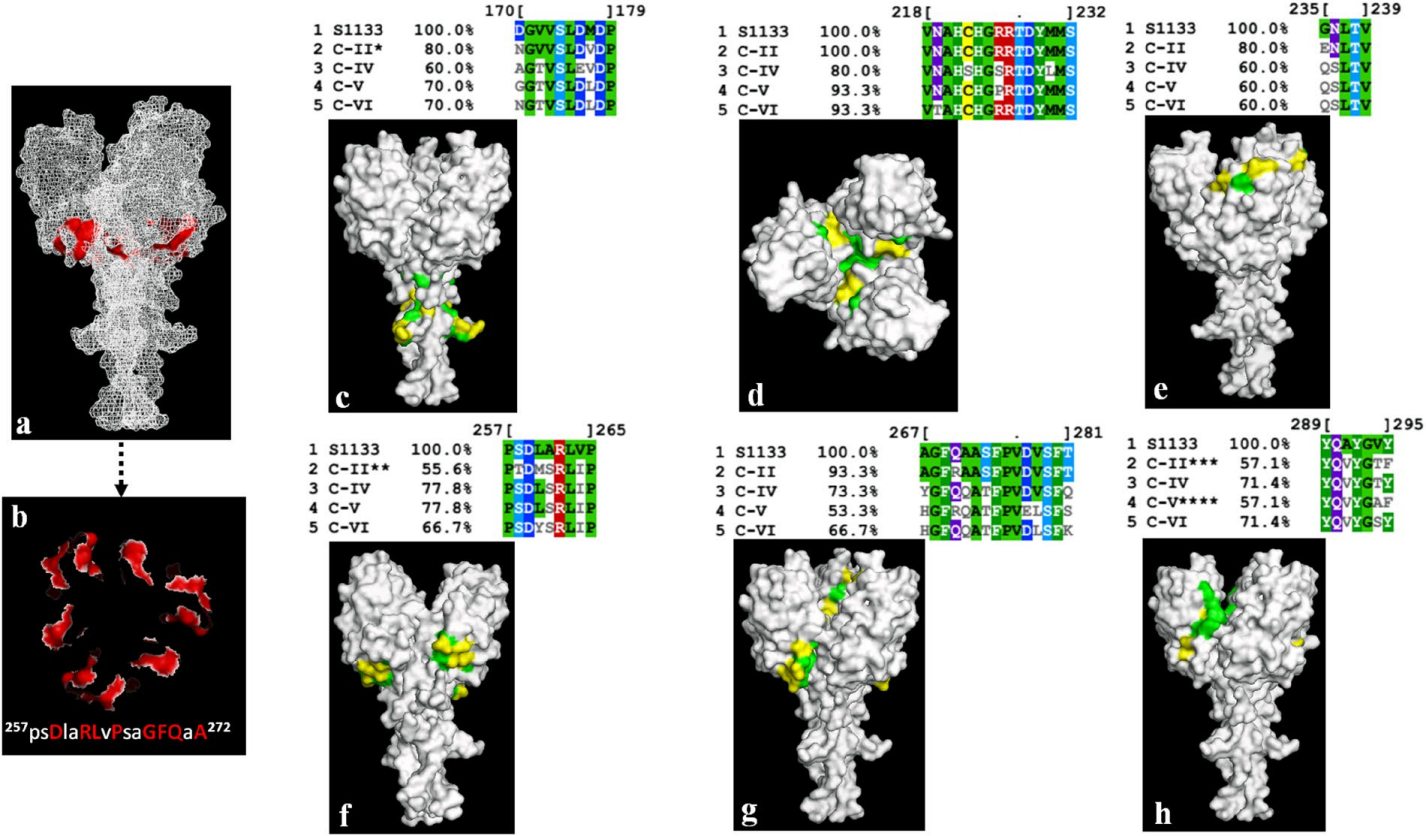
Conserved and variable regions of predicted antigenic epitopes of the Sigma C protein shown on the modelled Sigma C carboxy terminus receptor binding domain trimeric structure. (**a** and **b**) Conserved proposed receptor binding residues. The conserved residues are highlighted in red as “cartoons”. (**c** to **h**) The conserved amino acid residues on the predicted antigenic epitopes between the S1133 vaccine strain and our isolates which grouped into different cluster “C” groups are shown in green as “surface” on the carobxy terminus receptor binding domain model structure of the Sigma C protein. The variable regions are shown in yellow. *^194^DV substituted by ^194^VM, **^260^M substituted by I, ***^294^T substituted by N or A and ****^294^A substituted by N or D in some cases.

A blast search in PDB protein data bank using Sigma C protein sequences of our ARV isolates produced the S1133 Sigma C protein structure [PDB 2jjl]^10^ as the first hit and was chosen as a template structure. Superimposition of the Sigma C protein model structures of our isolates revealed similar topology as the head region of the Sigma C protein of the S1133 ARV strain (PDB 2jjl). The conformation in the amino terminal region (i.e. amino acids 120 to 150) was variable between the cluster groups. In the DE loop domain of the head region, amino acids ^259^D ^262^R^263^L^265^P^268^G^269^F^270^Q and ^272^A were fully conserved in all our isolates. The conserved residues are highlighted in red as cartoons in Fig. 8a and b. Comparison of six antigenic peptides predicted based on the S1133 Sigma C protein structure^15^ with antigenic peptides predicted for the Sigma C protein of our isolates demonstrated significant variability between the cluster groups and the S1133 strain (Fig. 8c to h). The conserved and variable amino acid residues for each peptide are presented as “surface” with green and yellow, respectively (Fig. 8c to h). The amino acid substitutions brought about a change in the secondary structures of the predicted antigenic epitopes in the modelled structures (Supplement Figure 4). Similarly, based on a blast search in the PDB data bank, Sigma 3 protein structure (PDB 1jmu) was used as a template structure to model the structures of the Sigma B protein of our isolates. Comparison of all the six predicted antigenic peptides of the Sigma B protein of the isolates revealed 100% conservation between the cluster groups. The conserved peptide amino acid residues are presented as “surface” with green in the secondary protein structure (Supplement Figure 5).

## Discussion

ARVs are important pathogens of poultry. The most identifiable problem directly associated with ARV is viral arthritis/tenosynovitis, which commonly affects broiler chickens^6, 16^. Since ARVs are resistant to heat, proteolytic enzymes, various disinfectants and a wide spectrum of pH, it is almost impossible to maintain poultry farms free from ARV infection^17^. Therefore, the most viable option to prevent the disease is the use of appropriate vaccines. The failure of conventional vaccines and the increased rate of diagnosis of the disease in the last five years are alarming indicating that the emerging pathogenic ARVs are becoming imminent threats to the broiler industry across North America. The effectiveness of a vaccine largely depends on the antigenic similarity between the vaccine and field strains. Thus, the present study was planned to gain knowledge on circulating ARV strains in Saskatchewan by isolating and characterizing viruses from the field cases of arthritis/tenosynovitis.

In this study, we observed a 98.3% prevalence of ARV infection based on ELISA similar to the ARV prevalence reported in Iran (98.3%)^18^, and higher than the prevalence reported in Romania (85.5%)^19^, Turkey (70.6 to 77.2%)^20^ and Nigeria (41%)^21^. The IDEXX ELISA kit plates are coated with a whole virus lysate and detect “total” antibody response, therefore the kit is not strain-specific and highly cross reactive to a wide range of pathogenic and non-pathogenic ARV strains. Since pathogenic ARVs mainly affect young broiler chickens and, the IDEXX ARV ELISA kit does not discriminate between pathogenic and non-pathogenic ARV infections, the high prevalence solely based on ELISA does not directly associate with disease conditions. However, the results indicate that ARV infection is ubiquitous in the commercial broiler chicken farms in Saskatchewan. To selectively detect specific antibodies produced against infections due to virulent strains of ARV, pathogenic ARV specific diagnostic kits have to be developed in the future.

We have isolated ARV from broiler chickens with lameness and histopathological lesions of tendinitis as in the previous reports^4, 15, 22^. As described earlier^4^, distinctive CPE typical of ARV was observed in the positive sample-inoculated LMH cells with fusion of the cells and characteristic syncytia formation. This fusogenic property of ARV is attributed to the p10 protein, which is expressed by the first open reading frame of the tricistronic S1 gene^16, 23^.

ARVs exhibit non-enveloped icosahedral structure with a double capsid layer and a central segmented double stranded RNA genome^24, 25^. Likewise, our isolates had identical features characteristic of ARV on negative staining TEM. In addition, indirect immuno-staining of the virus infected cells with anti-ARV specific antibody followed by confocal microscopy, and immunogold staining of ultrathin sectioned infected tendon tissues followed by TEM confirmed the virus isolates as ARVs.

As RT-PCR followed by RFLP analyses have been effectively employed by previous studies for detecting mutations and sequence polymorphisms in RNA virus isolates^26^, including ARVs^27^, we next investigated the genetic variability among the ARVs isolated from these field cases using these techniques. RFLP analyses of the Sigma C gene using *Dde*I, *Hinc*II, *Taq*aI, *Bcn*I, or *Hae*III restriction enzymes revealed different cleavage patterns indicating significant genetic heterogeneity between the current ARV isolates.

Molecular classification and genotypic relationship studies have helped in understanding the epidemiology, origin and evolution of emerging variant virus strains^13, 28^. Such studies on ARVs are mainly conducted using the sequence of the most variable cell attachment protein Sigma C^3, 4, 15, 28^, and only the representative part of the Sigma C gene is sufficient for the classification of virus isolates^13^. Since its isolation by Fahey and Crawley in 1954^29^, at least six genotypes of ARV are identified worldwide^3, 28^.

To gain greater insight into the genetic variability among the circulating ARVs, we next resorted to nucleotide sequencing and sequence data analysis. Based on the Sigma C protein sequence, our ARV field isolates were genetically diverse and clustered into four genotyping groups. They were also distinct from the Tunisian^28^ and the Israeli^15^ isolates that were closely related to the S1133 vaccine strain. This suggests that all our isolates were genetically quite distant from the vaccine and vaccine related field strains. Like the isolates from Germany, Netherlands and Taiwan^13^, our isolates were widely dispersed signifying the emergence of divergent pathogenic ARV genotypes in the region. Interestingly, we isolated ARVs that grouped under cluster IV in the year 2014, which were not detected in the year 2013 indicating rapid evolution of the virus in the region. Accumulation of mutations and multiple re-assortment events are a common phenomenon in segmented RNA viruses^30^, which may have possibly led to genetic and pathotype shifts resulting in the evolvement and the emergence of the new variant strains. Our findings, that none of the present isolates were clustered together with the reference vaccine strains and had only a 53% maximum amino acid sequence identity with the American S1133 vaccine strain, suggests that circulating ARV strains have evolved and diverged significantly compared to the vaccine strains.

Some of our field isolates were closely related to strains isolated from USA, Germany, Netherlands or Taiwan. Our isolates that grouped under Cluster IV had the highest Sigma C protein sequence identity with the most recent USA ARV isolates^4^ as compared to other cluster groups suggesting that the Cluster IV isolates might have the same epidemiological origin as the US isolates. However, since our isolates are genetically diverse and the vaccines prepared from ARV strains originating from countries except the USA are never used in Canada, it is difficult to exactly pin-point the evolutionary origin of these emerging variant strains and the correlation between them solely based on the Sigma C sequence.

Despite implementation of ARV vaccination programs, there has been frequent incidence of ARV associated disease outbreaks in all geographic locations in Canada and the USA since 2011/2012^4, 31^. The ARV vaccines used in Canada are based on strains that were isolated in the United States. Hence, the failure of ARV vaccination programs and increased incidence of ARV associated tenosynovitis outbreaks can be partially due to the infection of broilers with the emerging ARV subtypes that are genetically quite distant from the vaccine strains, which is supported by the inability of antibodies raised against ARV vaccine strain 2177 to cross-neutralize the current isolates. The discrepancy observed between the experimental failures to cross-neutralize the variant strains by antibodies induced by the vaccine strain and a certain degree of vaccine protection in the field might be attributed to a high heterogeneity of ARV strains in the field conditions. Though there is a certain degree of conservation in the Sigma-C protein between the vaccine and the variant strains, the highly variable regions that are exposed on the surface of the Sigma C protein determine pathogenicity and antigenic escape. Moreover, the ARV isolates from all the four cluster groups caused tendinitis/tenosynovitis in experimentally infected SPF birds indicating that our isolates are virulent variant strains. As reported by Kort *et al*.^27^, infection of broiler chickens with emerging variant ARV strains has the ability to evade immunity induced by the conventional reovirus vaccines resulting in the persistence of ARV associated disease in poultry flocks.

Structure based analysis of the head domain of the Sigma C protein of our isolates had similar topology with the S1133 Sigma C protein. The proposed receptor-binding residues in the DE-loop region of the head domain^10^ are fully conserved in all our isolates. The high variability among the predicted antigenic epitopes between the cluster groups and the S1133 vaccine strain explains the reasons why the currently used commercial vaccine strains are ineffective against the circulating emerging ARV strains. Unlike the Sigma C protein, the antigenic epitopes on the structure of the major structural Sigma B proteins of our isolates are highly conserved. Previous studies^11^ also suggest that Sigma B proteins carry group specific epitopes.

In conclusion, our study demonstrates that emerging ARV variants associated with viral arthritis/tenosynovitis are evolving and can evade immunity induced by commercial vaccine strains. Therefore, development of new vaccines tailored against the emerging pathogenic ARV variant strains is essential to minimize economic losses to the broiler chicken industry. Further studies like full genome sequencing and characterization and determining the mechanism of pathogenicity are required to gain better insights into the circulating strains of ARVs to develop efficient disease protective strategies.

## Methods

### Cells and media

Leghorn male hepatoma (LMH CRL-2177) cells (ATCC) were used for isolation/propagation of ARV isolates and for virus neutralization test (VNT). The growth medium used was DMEM-12 supplemented with 10% fetal calf serum, 20 mM HEPES and 2 mM L-glutamine (Life Technologies) and 50 mg/ml gentamicin. For propagation of virus stock, the same media was used without fetal calf serum.

### Enzyme Linked Immunosorbent Assay (ELISA)

Serum samples were collected from 59 broiler farms in Saskatchewan in 2014 at the time of slaughter (between the ages of 33 to 42 days). At farms where there were one or two flocks, only one flock was sampled, whereas at farms with more than three flocks, two flocks were sampled. Twenty serum samples were collected from each flock for a total of 1980 samples. Antibody titers were measured by using ARV specific enzyme-linked immunosorbent assay (ELISA) kit (Part Number: 99–09264, IDEXX Laboratories, Inc, Westbrook, Maine, USA) as per the manufacturer’s protocol. The ELISA plates are coated with whole virus lysate and detect total antibody response; therefore the kit is not strain specific. Briefly, samples were diluted 1 in 500 and 100 μL of diluted samples added to the wells of the ELISA microplate. After incubation for 30 minutes at room temperature the liquid content was removed and the plate was washed 5 times with 300 μL of distilled water. One-hundred μL of (goat) anti-chicken horseradish peroxidase conjugate was then added and the plate was incubated for 30 minutes at room temperature. The liquid content of wells was removed and plates were washed 5 times with 300 μL of distilled water. One-hundred μL of 3, 3′, 5, 5′-tetramethylbenzidine substrate solution was added and incubated for 15 minutes at room temperature. After incubation 100 μL of “Stop Solution” was dispensed and absorbance values were measured at 650 nm.

### Histopathology

Sections of affected gastrocnemius tendon tissues were fixed in 10% neutral buffered formalin. The fixed tissues were embedded in paraffin, sectioned at 5 μm thickness, stained with hematoxylin and eosin, and examined under a light microscope.

### Virus Isolation, propagation and plaque purification

Tendon tissues were collected from broiler chickens with arthritis/tenosynovitis from 25 broiler farms in Saskatchewan from 2013 to 2015. The samples were individually minced and homogenized in tubes with beads using a bullet blender storm-24 (Next Advance) for 10 min at a speed of 12. The homogenate was centrifuged at 3,000 rpm for 10 min and the supernatant was filtered through a 0.22 µm filter. The filtrate was diluted with media without serum and added onto LMH cells freshly grown on 6 well plates. Subsequently, the cells were observed for the appearance of reovirus specific cytopathic effect (CPE) over a period of seven days. Finally, the supernatants were collected from positive wells and passaged once before storing at −80 °C. Plaque purification was performed as previously described^32^.

### Virus Purification and Transmission Electron Microscopy (TEM)

Plaque purified ARV infected cell lysates were collected and freeze-thawed five times. The lysates were then incubated with 1.5 mL of sodium deoxycholate (5%) for 30 min at room temperature. Once the suspension became viscous, it was incubated with 2 M MgCl_2_ and DNase-I solution for 1 hr at 37 °C. Finally, the lysate was centrifuged at 3,500 rpm on a table top centrifuge for 15 min, and the supernatant was loaded on to CsCl density gradient (1.25/1.44 g/mL) cushion. After overnight centrifugation at 40,000 rpm at 10 °C, the viral bands were separated and stored for further application or negatively stained and visualized under transmission electron microscopy (TEM).

### Ultrathin sectioning, Immunogold staining and TEM

Immunogold staining of ultrathin sectioned tendon tissues were performed as described by Deshmukh *et al*., 1971^24^ with few modifications. Ultrathin sectioned tendon samples were blocked with 1% (w/v) Y-globulin free serum albumin three times, 10 min each. Subsequently, the samples were incubated with a 20 µl drop of anti-reovirus primary antibody diluted in blocking buffer for 1 hr at room temperature. The grids were rinsed 3 × 5 min by passing them through a series of drops of PBS and incubated on 20 µl drops of secondary antibody (gold conjugate) diluted in blocking buffer for 1 hr at room temperature. Subsequently, they were rinsed 3 × 5 min with PBS and washed with water and incubated with 20 µl drops of 2% aqueous uranyl acetate with a drop of triton X-100 for 20 min followed by 4 × 4 min washes in distilled water. After the final wash, the excess water was blotted with Kimwipes and the grids were incubated with Renold’s lead citrate for 10 min followed by 5 × 4 min washes with water. Finally, the samples were blotted with Kimwipes, put on grid grippers and sections were observed under electron microscopy.

### Indirect immunostaining and confocal microscopy

LMH cells grown on 2 well chamber slides were infected with individual reovirus isolate. At 24 hr or 48 hr post-infection, the cells were fixed with 3.7% paraformaldehyde and permeabilized with 0.05% triton X-100. The permeabilized cells were blocked with 0.2% gelatin, washed three times with 1X PBS and incubated with anti-reovirus polyclonal antibody (Abcam) at room temperature on a shaker for 1:30 hr. Subsequently, the cells were washed three times, 5 min each and incubated with goat anti-chicken Alexaflour 647 (Abcam) secondary antibody for 1 hr at room temperature. After washing and drying, the slides were mounted using Vectashield containing DAPI (Vector Laboratories) and visualized under a confocal microscope.

### Restriction enzyme fragment length polymorphism (RFLP)

The Sigma C protein encoded by S1 genome segment is a major antigenic determinant for ARVs^4^. Therefore, we carried out PCR-RFLP analyses of Sigma C to determine the genetic variability among the field isolates. Briefly, RNA was purified from infected LMH cells and RT-PCR was performed using a one-step RT-PCR kit (Qiagen). The PCR products were purified from agarose gel using GenJet gel purification kit (Fermentas). The purified DNA fragments were digested with either *Dde*I, *Hinc*II, *Taq*aI, *Bcn*I, or *Hae*III restriction enzymes. The enzymes were selected based on restriction enzyme sites on Sigma C DNA sequences of the isolates and previously published S1 gene sequences of ARV^27, 33^. The digested gene fragments were run on a vertical 10% DNA polyacrylamide gel, stained with ethidium bromide and imaged using a gel-doc (Biorad).

### RT-PCR, sequencing and phylogenetic tree analysis

A segment of the Sigma C gene of ARV was amplified by RT-PCR using specific primers. Briefly, total RNA was purified from virus infected cells using RNA purification kit (Qiagen) as per the company’s protocol followed by RT-PCR using a one-step RT-PCR kit (Qiagen) and forward/reverse degenerate primers^15^. The 960 bp PCR product(s) were run on a 0.8% agarose gel and purified using a gel purification kit (Qiagen) as per the company’s protocol. The DNA samples were then sequenced using Sigma C forward and reverse primers (Macrogen, South Korea). Assembly, sequence analysis and phylogenetic tree construction was performed using Geneious 9.1.2 software (Geneious, Inc). For phylogenetic tree analysis, Sigma C protein sequences of 63 reference ARV strains and variant sequences from around the world retrieved from Genbank were included (Supplement Table 2). Similarly, the Sigma B genes of the isolates were PCR amplified and sequenced using SigB F- 5′ TTCTGGAATGCACAGACAGCCTGGGA as forward and SigB R-5′ TTACCAACCACACTCCACA as reverse primers. The sequences were deposited in the GenBank (Supplement Table 3).

### Virus neutralization test (VNT)

Four novel variant ARV strains from genotypic cluster-II, IV, V and VI including ARV vaccine strain 2177, were tested against the sera obtained from the birds vaccinated with Reo 2177 ^®^ (Merck) in the VNT. Briefly, the heat inactivated sera samples were 2 fold serially diluted in a 96 well plate, and 60 µl of reovirus preparation (200 TCID_50_) was added to all wells except negative control wells. Subsequently, the virus-sera mixture was incubated at 37 °C for 1 hr before adding onto freshly seeded LMH cells (5 × 10^4^ cell/well) in another 96 well plate with 2.5% fetal calf serum. Virus infected and uninfected cells were used as positive and negative controls, respectively. The experiment was performed in triplicates for each virus tested.

### Pathogenicity experiment

Specific pathogen free (SPF) eggs were purchased (Sunrise Farms, Inc. USA), incubated and hatched. Day old chicks were divided in to five groups, 12 birds in each group. The groups were kept in separate isolation units in the Western College of Veterinary Medicine (WCVM) animal care unit and provided with food and water in ad libitum. Each group of birds were infected with a different strain of ARV isolate from each cluster group via the right footpad using 25 gauge needle. The 5th group was injected with saline and kept as a negative control. The birds were monitored three times a day and observed for development of clinical signs. Tendon samples were collected after birds were euthanized and histopathology and gold-immunostaining electron microscopy were performed. The animal experiment was approved by the University of Saskatchewan’s Animal Research Ethics Board (Approval #: AUP 20160010) and was conducted according to the Canadian Council on Animal Care (CCAC) guidelines.

### Comparative protein structure modelling and antigenicity prediction

The target amino acid sequence of each Sigma C or Sigma B protein from each isolate was blasted on the PDB protein data bank to get a template structure. A complete alignment of the template and target sequences was performed using ClustalX 2.1 software and the missing residues were removed from the template sequence. The three dimensional model structure of the target sequences was modelled (i.e 20 models per target sequence) using the MODELLER software^34^. The models with the lowest DOPE pseudo energy score were selected for further analysis. The model structures were visualized and analyzed using PyMOL PDB viewer (Pymol.org). Antigenic epitopes predicted for S1133 ARV strain^15^ were compared with antigenic sequences predicted for our isolates using MView(http://www.ebi.ac.uk/Tools/msa/mview/). In addition, the Sigma B antigenic epitopes were predicted using EMBOSS-antigenic (http://www.bioinformatics.nl/cgi-bin/emboss/antigenic) and Kolaskar and Tongaonkar antigenic prediction (http://imed.med.ucm.es/Tools/antigenic.pl). The predicted antigenic peptide sequences were compared using MView.

### Data Availability

The ARV Sigma C and Sigma B sequence data analyzed during the current study are available in the GenBank. GenBank accession numbers are provided in Tables 1, 2 and 3 in the supplementary information file.

## Electronic supplementary material

Supplementary Information
