# Supplementary material for: Phenotypic, genotypic and antigenic characterization of emerging avian reoviruses isolated from clinical cases of arthritis in broilers in Saskatchewan, Canada
Source: Sci Rep. 2017 Jun 15;7:3565. doi: 10.1038/s41598-017-02743-8 (PMC5472580; doi:10.1038/s41598-017-02743-8)
Supplement: Supplementary file 1 — Supplementary Information [file 41598_2017_2743_MOESM1_ESM.pdf]

Phenotypic, genotypic and antigenic characterization of emerging avian reoviruses isolated from clinical cases of arthritis in broilers in Saskatchewan, Canada

Authors: Lisanework E Ayalew, Ashish Gupta, Jenny Fricke, Khawaja Ashfaque Ahmed, Shelly Popowich, Betty Lockerbie, Suresh K Tikoo, Davor Ojkic, Susantha Gomis*

*Corresponding author: 52 Campus Drive, University of Saskatchewan, Saskatoon, Saskatchewan, Canada S7N 5B4. Phone #: +1-306-966-7299

E-mail address: susantha.gomis@usask.ca

Supplement Table 1. A list of ARV isolates and their Sigma C gene sequences deposited in GenBank

Isolate	Symptom	GenBank Accession #	Genotyping Cluster (Sigma C)
SK-R1	Tenosynovitis	KX855899	VI
SK-R2	Tenosynovitis	KX855900	VI
SK-R3	Tenosynovitis	KX855901	VI
SK-R4	Tenosynovitis	KX855902	VI
SK-R5	Tenosynovitis	KX855903	VI
SK-R6	Tenosynovitis	KX855904	VI
SK-R7	Tenosynovitis	KX855905	VI
SK-R8	Tenosynovitis	KX855906	VI
SK-R9	Tenosynovitis	KX855907	VI
SK-R10	Tenosynovitis	KX855908	V
SK-R11	Tenosynovitis	KX855909	V
SK-R12	Tenosynovitis	KX855910	V
SK-R13	Tenosynovitis	KX855911	IV
SK-R14	Tenosynovitis	KX855912	IV
SK-R15	Tenosynovitis	KX855913	IV
SK-R16	Tenosynovitis	KX855914	IV
SK-R17	Tenosynovitis	KX855915	IV
SK-R18	Tenosynovitis	KX855916	IV
SK-R19	Tenosynovitis	KX855917	IV
SK-R20	Tenosynovitis	KX855918	IV
SK-R21	Tenosynovitis	KX855919	IV
SK-R22	Tenosynovitis	KX855920	II
SK-R23	Tenosynovitis	KX855921	II
SK-R24	Tenosynovitis	KX855922	II
SK-R25	Tenosynovitis	KX855923	II
SK-R26	Tenosynovitis	KX855924	II
SK-R27	Tenosynovitis	KX855925	II
SK-R28	Tenosynovitis	KX855926	II
SK-R29	Tenosynovitis	KY026178	VI
SK-R30	Tenosynovitis	KY026179	II
SK-R31	Tenosynovitis	KY026180	VI
SK-R33	Tenosynovitis	KY026182	VI
SK-R34	Tenosynovitis	KY026183	V
SK-R35	Tenosynovitis	KY026184	II
SK-R36	Tenosynovitis	KY026185	II
SK-R37	Tenosynovitis	KY026186	VI
SK-R38	Tenosynovitis	KY026187	II

Supplement Table 2. A list of ARVs and Sigma C gene sequences retrieved from the GenBank for this study

Isolate	Country	Symptom	GenBank Accession #	Genotyping Cluster (Sigma C)
JR1	England	Unclear	EF122836.1	I
S1133	USA	Tenosynovitis	AF330703	I
TU5	Tunisia	MAS	HM751145	I
TU87	Tunisia	Unclear	HM751144	I
TU96	Tunisia	Tenosynovitis	HM751139	I
TU97.1	Tunisia	Tenosynovitis	HM751140	I
TU399	Tunisia	Tenosynovitis	HM751135	I
TU430	Tunisia	Tenosynovitis	HM751137	I
V.A.Vac	USA	Vaccine	EF122837	I
2408	USA	MAS	AF204945	I
601SI	Taiwan	Tenosynovitis	AF204947	I
1733	USA	Tenosynovitis	AF004857	I
VA-1	India	Vaccine	EU681255.1	I
ISR5220	Israel	Tenosynovitis	FJ793532	I
ISR5226	Israel	Tenosynovitis	FJ793547	I
ISR5225	Israel	Tenosynovitis	FJ793546	I
04769a/14	USA	Tenosynovitis	KP727766	I
04660/14	USA	Tenosynovitis	KR856959	I
01384/14	USA	Tenosynovitis	KR856956	I
04667/14	USA	Tenosynovitis	KP727767	I
04666/14	USA	Tenosynovitis	KP727768	I
ISR5233	Israel	Tenosynovitis	FJ793549	III
GEL12 98M	Germany	MAS	AF354225.1	I
601G	Taiwan	Tenosynovitis	AF297217	I
R2-TW	Taiwan	Tenosynovitis	AF297213	I
918	Taiwan	MAS	AF297215	II
916	Taiwan	MAS	AF297214	II
GA41560	USA	RSS	DQ872798	II
GA/12274	USA	RSS	JX983599	II
GEL13a98M	Germany	MAS	AF354226.1	II
05273a/14	USA	Tenosynovitis	KR856981	II
05273b/14	USA	Tenosynovitis	KR856982	II
04455/13	USA	Tenosynovitis	KP727778	II
01382/14	USA	Tenosynovitis	KR856980	II
05287/14	USA	Tenosynovitis	KR856986	II
GA/12355	USA	RSS	JX983602	II
GEL 13B98M	Germany	MAS	AF354227.1	III
ISR5233	Israel	Tenosynovitis	FJ793549	III
GA41565	USA	RSS	DQ872799.1	III
42563-4	USA	RSS	DQ872801.1	III
28505a/11	USA	Tenosynovitis	KP727786	III
28505b/11	USA	Tenosynovitis	KR856990	III
22790/11	USA	Tenosynovitis	KP727787	III
07634/14	USA	Tenosynovitis	KR856992	III
28439/11	USA	Tenosynovitis	KR856989	III
GEL05 97M	Germany	MAS	AF354223	IV
NLA13 96T	Netherlands	Tenosynovitis	AF354228	IV
NLI12 96M	Netherlands	MAS	AF354230	IV
GEL03 97T	Germany	Tenosynovitis	AF354222	IV
GEL01 96T	Germany	Tenosynovitis	AF354221	IV

02807/14	USA	Tenosynovitis	KP727807	IV
03795/14	USA	Tenosynovitis	KP727805	IV
04870/14	USA	Tenosynovitis	KP727806	IV
05573/12	USA	Tenosynovitis	KP727800	IV
05907/14	USA	Tenosynovitis	KR857002	IV
1017-1	Taiwan	MAS	AF297216	V
03349/14	USA	Tenosynovitis	KR856994	V
04314/14	USA	Tenosynovitis	KR856995	V
05682/12	USA	Tenosynovitis	KP727791	V
12323/13	USA	Tenosynovitis	KP727793	V
GEL10 97M	Germany	MAS	AF354219	VI
RAM-1	Australia	Healthy	L38502	VI
SOM-4	Australia	Unclear	L07069	VI

Supplement Table 3. A list of ARV isolates and their Sigma B gene sequences deposited in GenBank

Isolate	Symptom	GenBank Accession #	Genotyping Cluster (Sigma C)
SigB_SK-R1	Tenosynovitis	KY633770	VI
SigB_SK-R2	Tenosynovitis	KY633771	VI
SigB_SK-R3	Tenosynovitis	KY633772	VI
SigB_SK-R5	Tenosynovitis	KY633773	VI
SigB_SK-R6	Tenosynovitis	KY633774	VI
SigB_SK-R7	Tenosynovitis	KY633775	VI
SigB_SK-R9	Tenosynovitis	KY633776	VI
SigB_SK-R10	Tenosynovitis	KY633777	V
SigB_SK-R12	Tenosynovitis	KY633778	V
SigB_SK-R13	Tenosynovitis	KY633779	IV
SigB_SK-R15	Tenosynovitis	KY633780	IV
SigB_SK-R16	Tenosynovitis	KY633781	IV
SigB_SK-R18	Tenosynovitis	KY633782	IV
SigB_SK-R20	Tenosynovitis	KY633783	IV
SigB_SK-R22	Tenosynovitis	KY633784	II
SigB_SK-R26	Tenosynovitis	KY633785	II
SigB_SK-R27	Tenosynovitis	KY633786	II

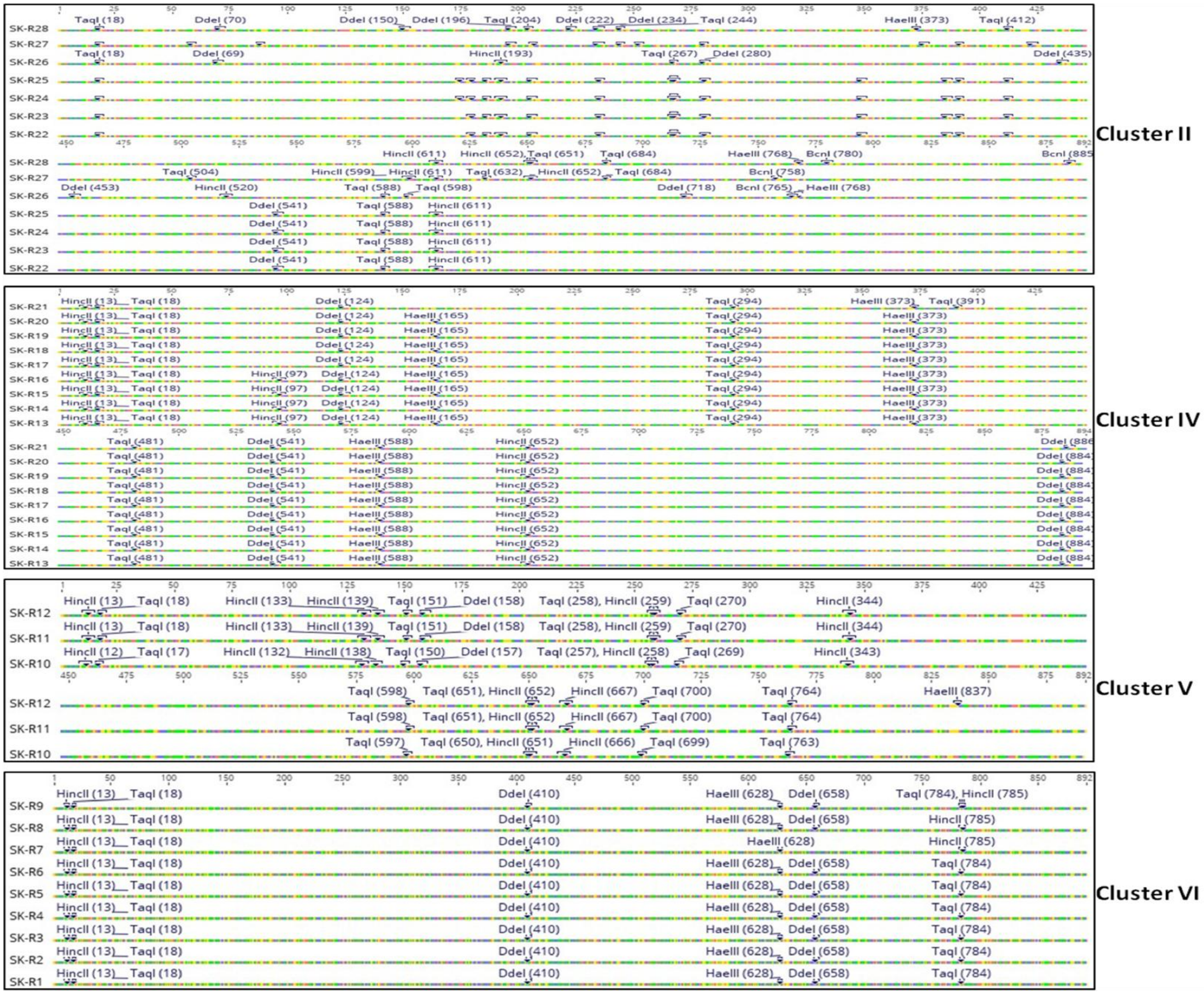

Supplement Fig 1. Mapping of *Dde*I, *Hinc*II, *Taq*aI, *Bcn*I and *Hae*III restriction enzyme sites on Sigma C DNA sequences of the ARV isolates.

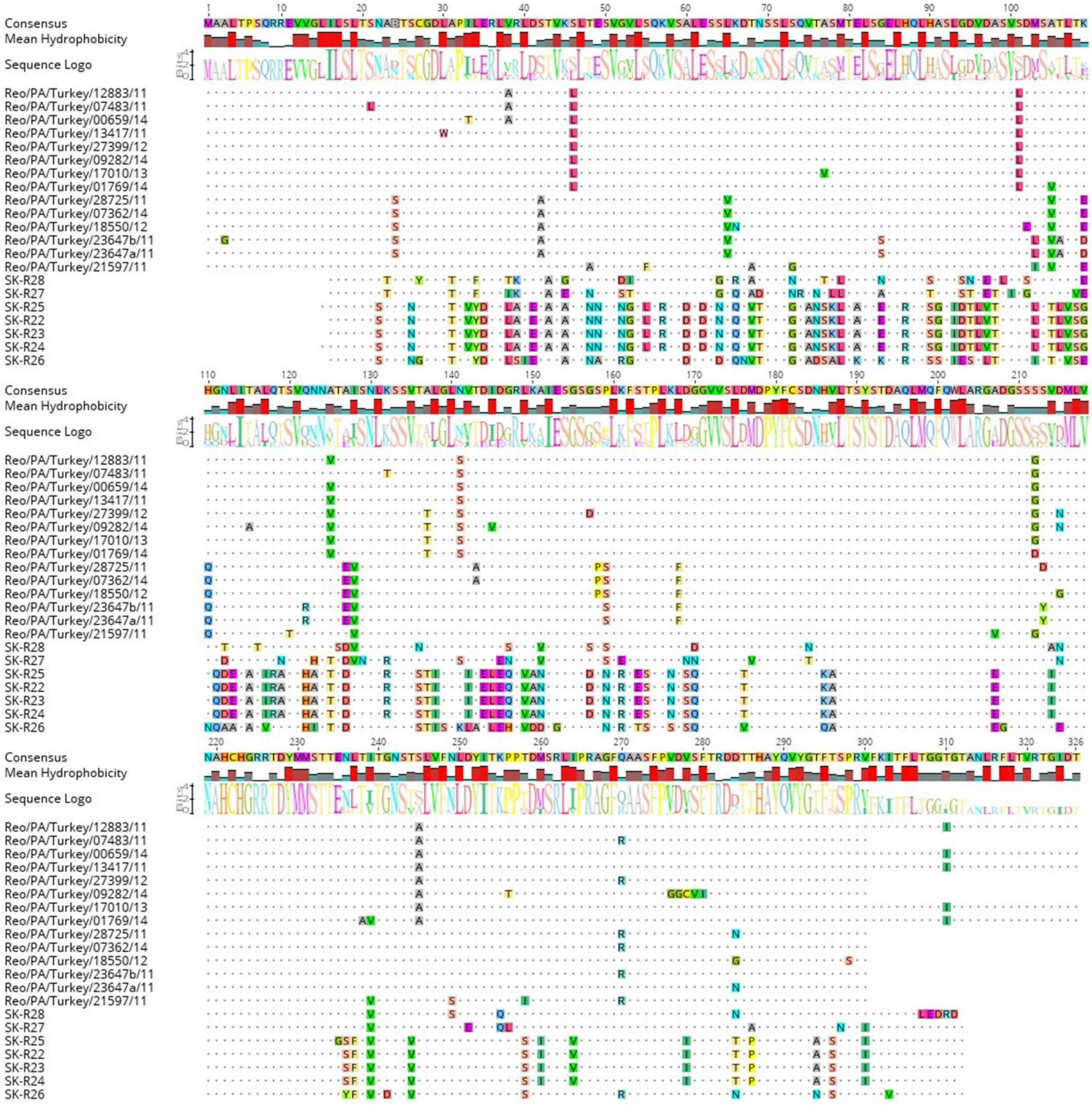

Supplement Fig 2. A multiple amino acid sequence alignment of the Sigma C protein sequences of our ARV isolates and the previously reported Turkey reovirus isolates from the USA that grouped under Cluster-II (Lu et al., 2015). The alignment was made by a ClustalW alignment with BLOSUM cost matrix technique. Disagreements to the consensus sequence are highlighted.

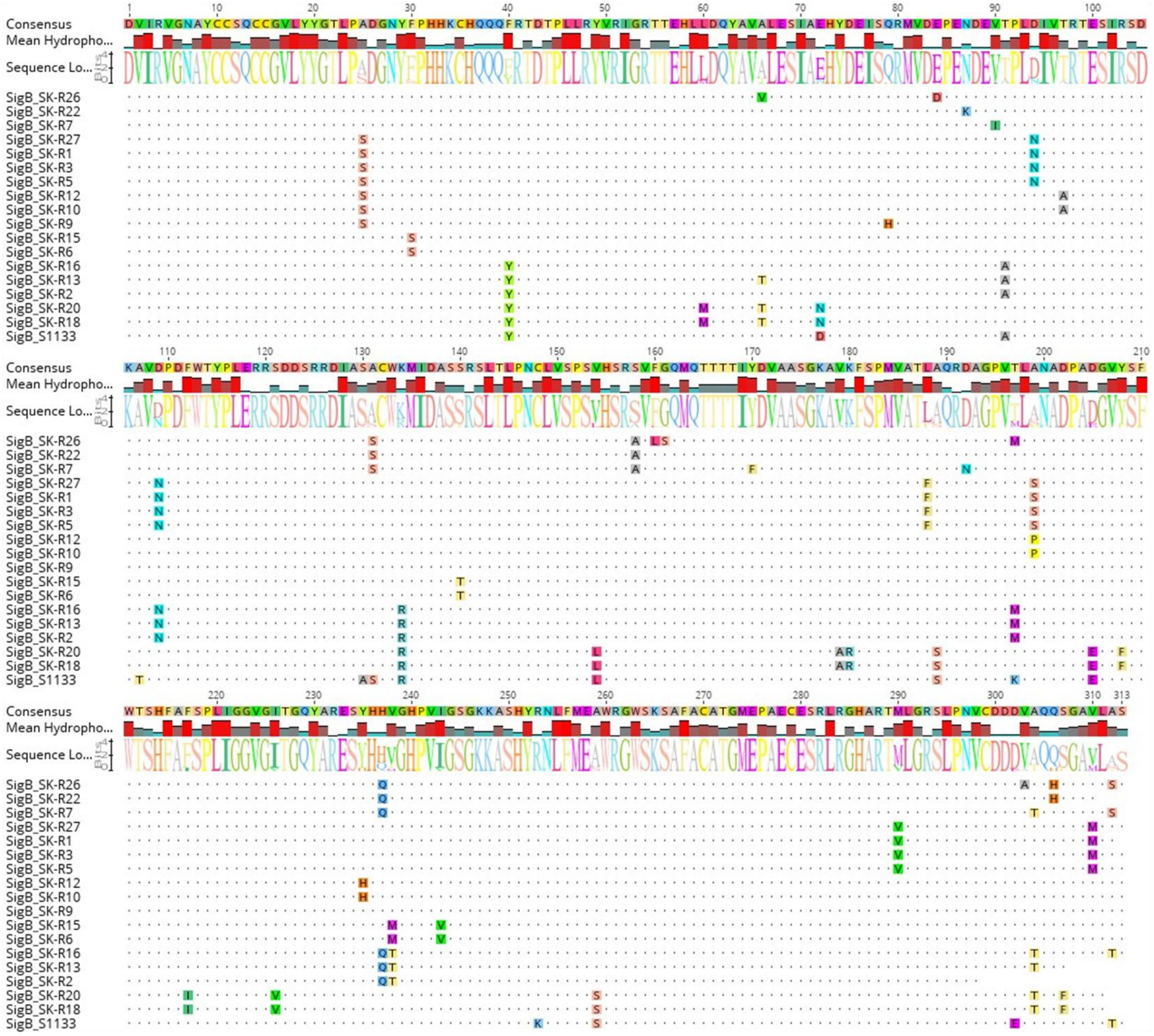

Supplement Fig 3. A multiple amino acid sequence alignment of the Sigma B protein sequences of the S1133 vaccine strain and our ARV isolates. The alignment was made by a ClustalW alignment with BLOSUM cost matrix technique. Disagreements to the consensus sequence are highlighted.

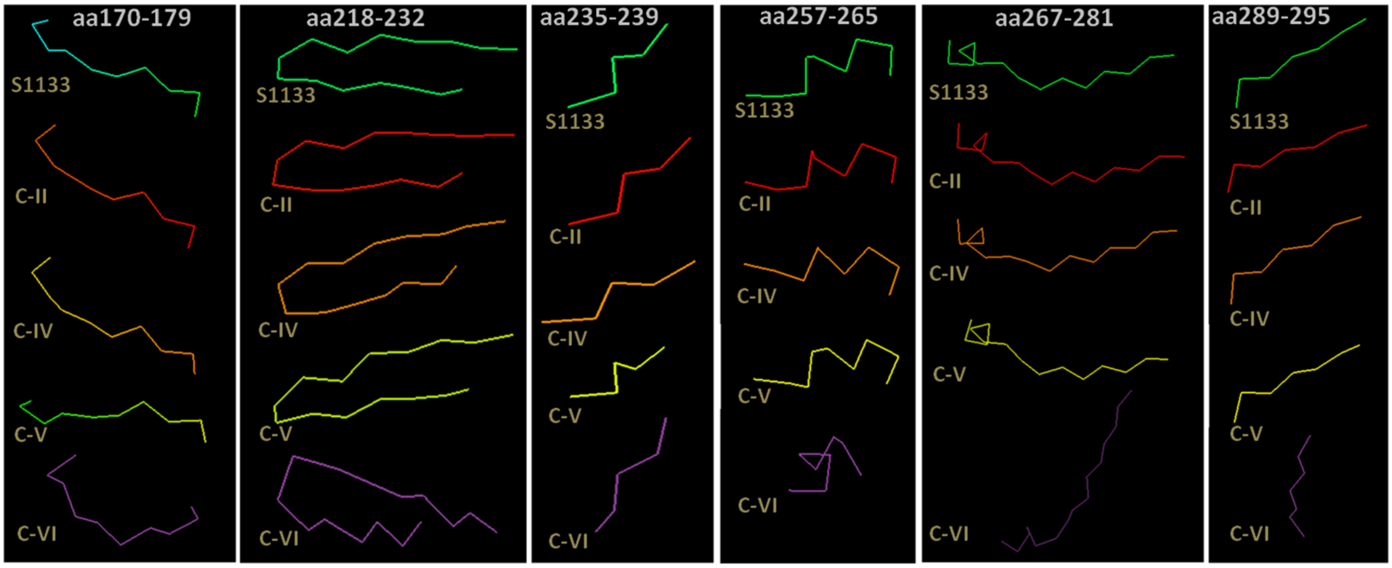

Supplement Fig 4. Secondary structures of the indicated predicted antigenic epitopes on the head domain of the Sigma C protein of S1133 vaccine strain and representative ARV isolates from the different cluster groups. The structures were visualized using PyMol software and presented as "ribbon" in the same orientation after protein structure alignment. "C"=Cluster.

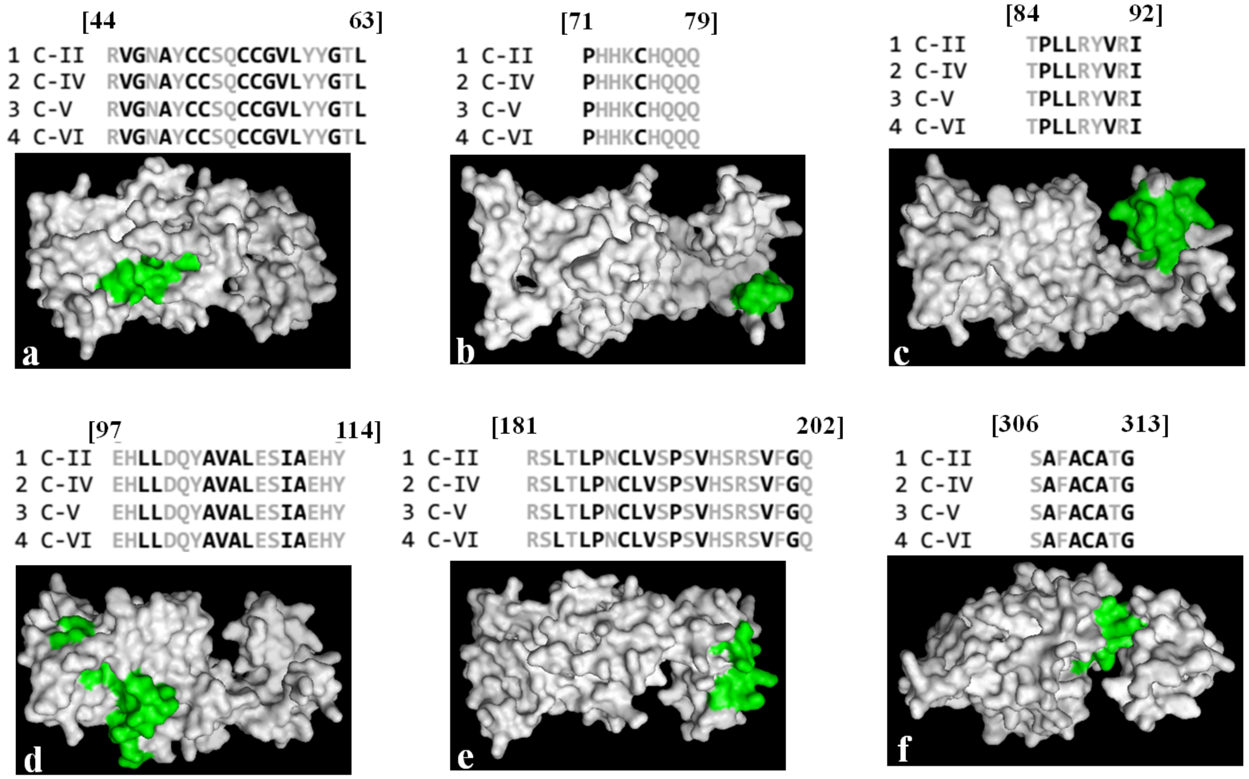

Supplement Fig 5. Predicted antigenic epitopes of the Sigma B protein shown on the modelled Sigma B secondary structure. (a to f) The conserved amino acid residues on the indicated predicted antigenic epitopes of the field isolates which grouped into different Cluster [C] groups are shown in green as "surface".
